# Early Bunyavirus-Host Cell Interactions

**DOI:** 10.3390/v8050143

**Published:** 2016-05-24

**Authors:** Amelina Albornoz, Anja B. Hoffmann, Pierre-Yves Lozach, Nicole D. Tischler

**Affiliations:** 1 Molecular Virology Laboratory, Fundación Ciencia & Vida, Av. Zañartu 1482, 7780272 Santiago, Chile; aalbornoz@cienciavida.org; 2 CellNetworks—Cluster of Excellence and Department of Infectious Diseases, Virology, University Hospital Heidelberg, Im Neuenheimer Feld 324, 69120 Heidelberg, Germany; anja.hoffmann@uni-heidelberg.de

**Keywords:** bunyavirus, cell entry, endocytosis, hantavirus, RNA virus, tospovirus, virus membrane fusion, virus receptor

## Abstract

The *Bunyaviridae* is the largest family of RNA viruses, with over 350 members worldwide. Several of these viruses cause severe diseases in livestock and humans. With an increasing number and frequency of outbreaks, bunyaviruses represent a growing threat to public health and agricultural productivity globally. Yet, the receptors, cellular factors and endocytic pathways used by these emerging pathogens to infect cells remain largely uncharacterized. The focus of this review is on the early steps of bunyavirus infection, from virus binding to penetration from endosomes. We address current knowledge and advances for members from each genus in the *Bunyaviridae* family regarding virus receptors, uptake, intracellular trafficking and fusion.

## 1. Introduction

The *Bunyaviridae* is a large family of RNA viruses, which comprises five genera (*Hantavirus*, *Nairovirus*, *Orthobunyavirus*, *Phlebovirus* and *Tospovirus*) [1]. With over 350 identified isolates distributed worldwide, these viruses represent a global threat to livestock, agricultural productivity and human public health. Many cause serious diseases with high mortality rates in domestic animals and humans, such as fatal hepatitis, encephalitis and hemorrhagic fever. Bunyaviruses are unique in the way they infect a large range of hosts, including vertebrates, invertebrates and plants. Recently, novel genera in the family have been proposed based on the identification of new bunyavirus members. However their host range has not yet been determined [2]. The increasing frequency of bunyavirus outbreaks over the last decade makes these viruses potential emerging agents of disease. No vaccines or treatments are currently approved for human use. Some are classified as potential biological weapons and listed as high-priority pathogens by the World Health Organization.

Most of the available information on bunyaviruses comes from studies of a limited number of isolates. However, it is apparent that there is a wide variety of viruses, vectors, hosts, diseases and geographical distributions. Hantaviruses are harbored in small mammals like rodents, shrews, moles, and bats, and are mainly transmitted to humans through inhalation of contaminated aerosols from the feces of infected rodents [3]. The other known bunyaviruses are all arthropod-borne viruses, which for convenience will be referred to as arbo-bunyaviruses; orthobunyaviruses, nairoviruses and phleboviruses spread to vertebrates by blood-feeding arthropods [4,5,6], while tospoviruses are plant-specific and are transmitted via non-hematophagous vectors, namely thrips [7]. For a more complete picture of bunyaviruses, we recommend recent books and reviews [1,2,3,4,5,6,7,8,9,10].

The diversity among the *Bunyaviridae* family is also manifested at the cellular and molecular levels in the genomic organization, virion structure, tropism, cellular receptors and cell entry. In this review, we address current knowledge and advances regarding early bunyavirus-host cell interactions, from virus binding to penetration into the cytosol.

## 2. Bunyavirus Genome Organization and Virion Structure

**Figure 1:**
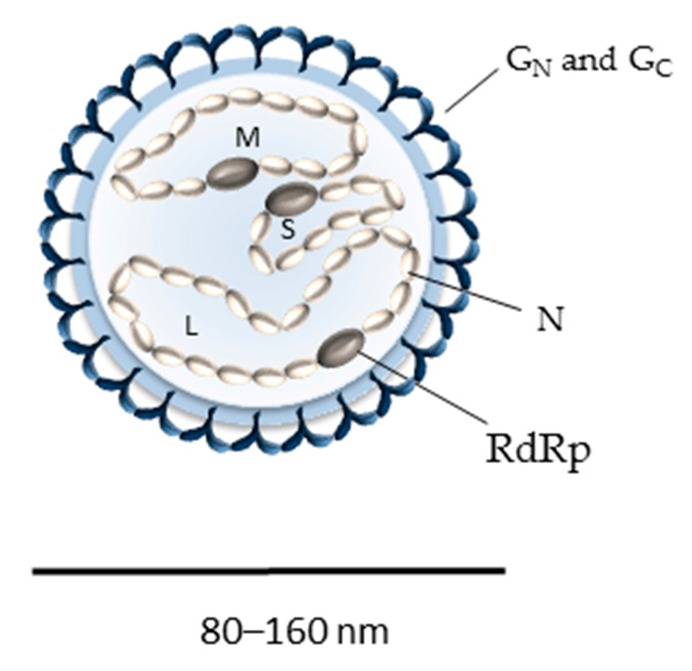
Schematic representation of a bunyavirus particle. The three viral genomic segments are termed according to their size: S (small), M (medium) and L (large). Abbreviations: G_N_: glycoprotein G_N_; G_C_: glycoprotein G_C_; N: nucleoprotein; RdRp: RNA-dependent RNA polymerase.

**Figure 2:**
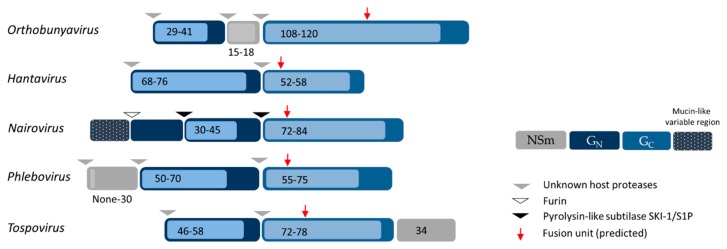
Schematic representation of bunyavirus G_N_ and G_C_ precursor glycoprotein sequences of each genus. Light and dark colored boxes indicate the smallest and highest molecular weight (kDa) of each protein in each genus, respectively. Arrow heads indicate the proteolytic cleavage sites within the glycoprotein precursor by host proteases [32,33]. Red arrows show the localization of the fusion peptide for each genus based on the crystal structure obtained from Rift Valley fever virus Gc [34] and on bioinformatics predictions and biochemical analysis of the glycoproteins from the orthobunyavirus La Crosse, the hantavirus Andes, the nairovirus Crimean-Congo hemorrhagic fever and the tospovirus tomato spotted wilt [35,36,37].

Bunyaviruses are enveloped with a tri-segmented single-stranded RNA genome, which replicates in the cytosol [1]. The three viral RNA segments code for a minimum of four structural proteins in a negative-sense orientation (Figure 1) [1]. The largest genomic RNA segment (L) encodes the RNA-dependent RNA polymerase L, which is required for the initiation of viral replication after the virus genome is released into the cytosol. The medium virus RNA segment (M) codes for a precursor polypeptide that is further processed into two envelope glycoproteins, G_N_ and G_C_, in the endoplasmic reticulum (ER) or Golgi apparatus (Figure 2), from where virions acquire their lipid bilayer membrane and assemble [1,11]. The precise location and mechanisms for the glycoprotein maturation and virus budding in the ER-Golgi machinery can differ among bunyavirus isolates and cell types and very often remain to be defined. The smallest segment (S) encodes the nucleoprotein N, which associates with the viral RNA genome and together with the viral polymerase L constitutes the pseudo-helical ribonucleoproteins (RNPs) [1]. Bunyaviruses do not possess any classical matrix protein or rigid inner structure. The N protein thus has an important role in protecting the viral genetic information. In the past five years, the crystal structure of N has been solved for several bunyavirus members, providing new insights into the mechanism of RNP assembly and showing some distinctions in the N proteins among the different genera [12,13,14,15,16,17,18,19,20,21,22,23,24,25,26,27]. Bunyaviruses also encode some non-structural proteins [28,29,30,31], but thus far, none have been found to be involved in virus entry and, therefore, will not be discussed here.

**Figure 3:**
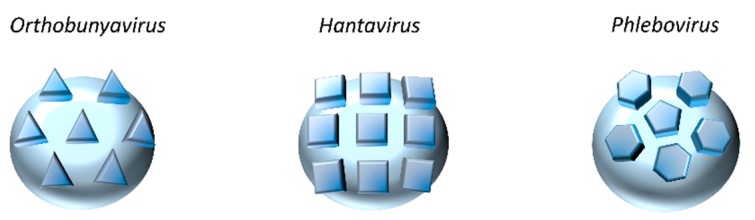
Schematic representation of the bunyavirus G_N_ and G_C_ glycoprotein arrangement on the surface of viral particles. The symmetries shown here were obtained by cryo-electron tomography and image reconstruction from Bunyamwera virus (*Orthobunyavirus*, left panel), Tula virus (TULV) and Hantaan virus (HTNV) (*Hantavirus*, middle panel), as well as Rift Valley fever (RVFV) and Uukuniemi (UUKV) viruses (*Phlebovirus*, right panel) [39,40,41,42,43,44,45]. Images were adapted from [44].

On particles, the two envelope glycoproteins G_N_ and G_C_ are responsible for virus attachment to target cells and acid-activated penetration [8,38]. Electron micrographs of bunyaviruses show particles that are roughly spherical, heterogeneous in size with an average diameter of 80–160 nm and with spike-like projections of 5–10 nm composed of G_N_ and G_C_ heteromultimers [1]. Recent cryo-electron tomography studies confirmed the high degree of pleomorphism previously observed for bunyaviruses [39,40,41,42,43,44,45]. Ultrastructural analyses of the phleboviruses Rift Valley fever (RVFV) and Uukuniemi (UUKV) revealed that the most regular particles exhibited surface glycoprotein protrusions arranged on an icosahedral lattice, with an atypical T = 12 triangulation [39,40,41,43]. In contrast, tomography data obtained for the orthobunyavirus Bunyamwera displayed non-icosahedral viral particles with glycoprotein spikes exhibiting a unique tripod-like arrangement, while spikes from hantavirus glycoproteins arrange with local symmetry into tetramers (Figure 3) [42,44,45].

## 3. Receptors for Arbo-Bunyaviruses in Mammalian Hosts

During natural transmission to mammalian hosts, arbo-bunyaviruses are introduced into the skin dermis by infected arthropods. Due to their presence in the anatomical site of initial infection, dermal macrophages and dendritic cells (DCs) are among the first cells to encounter the incoming viruses [8]. To establish infection and replicate, viruses need to gain access to the intracellular environment. This very first step is strictly dependent on surface-exposed cellular receptors that include proteins, lipids and glycans and to which virus particles bind [46,47]. Some surface receptors can mediate virus entry into cells, without the requirement of additional co-receptors and molecules. Alternatively, some primary receptors limit the free diffusion of viral particles and/or promote interactions with secondary receptor complexes, which are responsible for virus entry into the cytoplasm [46,48]. When viruses rely on many cellular surface factors for entry and infection, the primary receptor is often referred to as the attachment factor. Only a few surface attachment factors and receptors have been reported for bunyaviruses, and very often, their role in cell entry remains to be uncovered.

Virus-receptor interactions are often specific and multivalent. Binding to multiple receptor molecules clustered within microdomains can enhance the avidity of low-affinity interactions [46]. Polysaccharides on glycoproteins, as well as glycolipids in the extracellular matrix found at the surface of most mammalian cells are highly polar structures. They can serve as a first docking site for many viruses, including bunyaviruses, through electrostatic interactions, which are in general of low affinity [48,49]. Glycosaminoglycans (GAGs), such as heparan sulfate, have been shown to promote infection by RVFV and another phlebovirus, Toscana virus (TOSV) [50,51,52]. When heparin was used to compete with GAGs on the cell surface, infection by both viruses was significantly reduced. Similar results were obtained when heparan sulfate molecules were removed from cells by enzymatic digestion prior to being exposed to these viruses. Furthermore, cells deficient in the synthesis of heparan sulfate were shown to be less sensitive to RVFV infection [50,52]. Interestingly, glycoproteins from the cell-cultured RVFV used in one of these studies did not show any distinction in basic amino acids with those from the virus found in the serum or organs of infected animals [50]. This suggests that the heparan sulfate-dependence of RVFV does not seem to result from virus culture adaptation. However, the fact that cells lacking GAGs on their surface remain sensitive to infection, even at lower levels, indicates that these viruses can use alternative receptors to attach to and enter cells.

Recent work has shown that RVFV and UUKV target and infect DCs by subverting the C-type lectin Dendritic Cell-Specific Intercellular adhesion molecule-3-Grabbing Non-integrin (DC-SIGN; also known as CD209) [53]. In the presence of neutralizing antibodies, dermal DCs remained resistant to RVFV and UUKV infection [53]. When DC-SIGN was expressed at the surface of cells, which are usually poorly infected by bunyaviruses, a significant fraction of cells was infected by the Germiston orthobunyavirus and by many phleboviruses, including RVFV, UUKV, Punta Toro and TOSV [53]. The list of proposed bunyaviruses that are able to use DC-SIGN has since been extended (Table 1). Recent studies have shown that the lectin enhances infection by rhabdoviral particles pseudotyped with the glycoproteins of the orthobunyavirus La Crosse (LACV) and with those of severe fever with thrombocytopenia syndrome virus (SFTSV), an important emerging human tick-borne phlebovirus pathogen [54]. A similar approach has recently been used to investigate the role of DC-SIGN in infection by the Crimean-Congo hemorrhagic fever virus (CCHFV) [55], a tick-borne nairovirus that infects endothelial cells and macrophages, but also dermal-like DCs [56,57,58]. DC-SIGN provides an interesting bridge between arbo-bunyaviruses amplified in arthropod vectors and initial infection in humans. This immune receptor is: (1) expressed on immature dermal DCs, which are present in the anatomical site of virus transmission; and (2) specialized in capturing pathogens with an *N*-glycan coat of high-mannose residues, such as those on the glycoproteins of virions derived from insects [8,59]. For these reasons, interactions between DC-SIGN and insect-borne pathogens are thought to be the most relevant, although several studies have suggested a role for this lectin in infection by various microbes that are not transmitted by arthropods [59]. With a lower extension, DC-SIGN is also expressed on alveolar macrophages and DCs in the lungs [60] and may represent an interesting receptor candidate for aerosol-transmitted bunyaviruses. So far, hantavirus-DC-SIGN interactions remain to be investigated.

In addition to DC-SIGN, the phleboviruses RVFV, TOSV and UUKV have been shown to subvert Liver-Specific Intercellular adhesion molecule-3-Grabbing Non-integrin (L-SIGN) [62], a closely-related C-type lectin expressed in liver sinusoidal endothelial cells [59]. Others have established that rhabdoviral particles pseudotyped with the envelope glycoproteins of SFTSV, but not with those of RVFV and LACV, can subvert L-SIGN [54]. The engagement of multiple viral glycoproteins by homo-tetrameric lectins is critical for high-avidity interactions between the closely related C-type lectin DC-SIGN and the arbovirus dengue from the *Flaviviridae* family [73]. Rhabdoviruses and bunyaviruses differ significantly with regard to the assembly and maturation of viral progeny. The structural organization of *N*-glycans on their surface may not exactly reflect the organization on RVFV and LACV viral particles, which in turn could favor less efficient virus binding to C-type lectins and infection. It is tempting to postulate that by acting as an attachment receptor on liver sinusoidal endothelial cells, L-SIGN plays a role in the liver tropism of some bunyaviruses.

Nucleolin has been identified as a potential binding factor for the nairovirus CCHFV [63]. This factor has been identified following a strategy combining co-immunoprecipitation with a G_C_ ectodomain fragment as bait and mass spectrometry analysis. However, there is currently no evidence of surface nucleolin as an entry factor for CCHFV, although the protein was shown to be present on the surface of cells susceptible to the virus. Following a similar strategy, but instead using the G_N_ ectodomain as bait, it has been proposed that the non-muscle myosin heavy chain IIA (NMMHC-IIA) is involved in the infectious entry of SFTSV [61]. NMMHC-IIA is an intracellular protein that has been shown to reach the cell surface of human umbilical vein endothelial cells (HUVECs) and Vero cells [61]. Silencing of the NMMHC-IIA gene in HUVECs resulted in a decrease in SFTSV infection, while ectopic expression of NMMHC-IIA in HeLa cells, which lack the protein expression, but are sensitive to infection, resulted in a ~20-fold increased sensitivity to the virus [61]. It remains unknown whether this protein serves as an entry receptor or merely as an attachment factor. Recently, SFTSV was seen in secreted vesicles positive for CD63, a cellular marker associated with extracellular vesicles [74]. The virions within these vesicles were efficiently delivered to uninfected cells. This is the first evidence for bunyaviruses that an isolate hijacks the exocytic machinery for receptor-independent transmission and entry into host cells.

## 4. Receptors for Plant-Specific Bunyaviruses

The fact that tospoviruses are plant-specific sets these bunyaviruses apart from others. Little is known about the initial steps of tospovirus infection, both in arthropod vectors and plants. The tomato spotted wilt virus (TSWV), which is vectored by the western flower thrips *Frankliniella occidentalis*, is by far the most investigated tospovirus pathogen-vector system. TSWV represents a threat to agricultural productivity in the Southwestern United States, and now in South America, Europe and Australia, where the thrips *F.* *occidentalis* has recently spread [75,76]. The tospovirus glycoproteins are thought to have been conserved during evolution only to disseminate the virus in arthropod vector populations, but not in plants. Mutations in the TSWV glycoproteins that make the thrips *F.* *occidentalis* resistant to infection do not affect the spread of the virus in plant cells [77,78].

In insects, TSWV glycoproteins were found to interact with a protein of 50 kDa expressed in the larval midguts of *F. occidentalis*, the function of which remains unknown [65]. The 50-kDa protein was recognized by anti-idiotypic antibodies against G_N_ and G_C_ [65], though only the latter blocked infection [64]. Interestingly, adult thrips seem to lose the expression of this 50-kDa protein, and it is tempting to correlate the expression of the protein with the capacity of TSWV to infect larval, but not adult thrips through the midgut barrier [79,80,81].

In plants, tospoviruses are believed to propagate by active transport under the form of non-enveloped viral RNP structures through cell wall-embedded pores, the plasmodesmata [82]. The non-structural protein NSm of TSWV appears to be an important player in RNP cell-to-cell transfer in plants [83,84]. NSm has been shown to interact with a protein located on both orifices of the plasmodesmata pores in *Arabidopsis thaliana*, the At-4/1 protein [85]. Such plasmodesmial proteins are believed to have receptor-like properties for TSWV [86]. However, no direct evidence has been reported so far of At-4/1’s role in cell-to-cell TSWV spread. Not much is known about tospovirus receptors and early interactions with host cells.

## 5. Receptors for Aerosol-Transmitted Bunyaviruses: How Hantaviruses Target Cells

The aerosol transmission of hantaviruses to humans implies that upon inhalation, these bunyaviruses encounter the epithelium of the lung. In the terminal lung alveoli, epithelial cells are tightly attached to endothelial cells derived from a capillary network, with the particularity that their basement membranes are fused into a single layer, thereby forming a thin air-blood barrier [87]. Hantavirus infection of human endothelial cells causes dramatic changes in the barrier function of the endothelium [3,88,89,90]. As a consequence, increased capillary permeability and vascular leakage associated with pulmonary edema or hemorrhagic fevers are often observed in infected patients [91,92,93]. A substantial amount of work has been done to identify potential cellular factors involved in hantavirus infection of endothelial cells. Natural ligands of endothelial cell surface receptors, including vitronectin and fibronectin, but not heparin and laminin, were found to antagonize hantavirus entry [67]. Screening antibodies against different integrin subunits serving as receptors for vitronectin and fibronectin [94] revealed that anti-β1 integrin antibodies specifically impair the infection of Vero cells by the Prospect Hill (PHV) and Tula (TULV) viruses, while anti-β3 integrin antibodies reduced by 60%–70% the sensitivity of these cells to Hantaan virus (HTNV), Andes virus (ANDV) and other pathogenic hantaviruses (Table 1) [67,68]. When the human β3 integrin was overexpressed as heterodimers with the α_IIb_ or α_V_ subunits in CHO cells, a 20–30-fold increase in infection was observed for the Sin Nombre (SNV) and New York-1 virus (NY-1V), though these cells are otherwise not very sensitive to hantaviruses [67].

Integrins and endothelial cells represent an interesting model to explain the molecular and cellular basis of the pathogenesis associated with hantavirus infections in humans [88,89,90]. Pathogenic NY-1V and HTNV were shown to bind β3 integrins through a specific N-terminal PSI (plexin-semaphorin-integrin) domain [95]. This domain is only exposed and accessible when the receptor adapts an inactive conformation in which it exhibits low affinity to its ligand [96]. In this study, the aspartate in position 39 of human β3 integrin was found to be critical for promoting the infection of CHO cells. Interestingly, the murine β3 integrin carries an asparagine at position 39 instead and is unable to mediate infection unless it is substituted by aspartate [95]. This suggests that β3 integrin promotes infection by these viruses in humans, but may not do so in mice. In this regard, it remains to be determined whether integrins from natural rodent reservoirs may promote infection.

Epithelial and endothelial cells form polarized monolayers, an aspect that is often neglected in *in vitro* investigations of viral cell entry into different cell lines. The endothelial cell polarity can impact at large the distribution of the surface receptors, as well as the global organization of the exocytic-endocytic machinery and, therefore, the virus entry program [97,98,99]. For instance, β3 integrin seems to be mainly located on the basolateral side of polarized epithelial cells, allowing attachment to the extracellular matrix [100,101]. However, it has also been found on apical surfaces, where it detects soluble ligands and circulating cells in luminal compartments [102,103,104,105]. Efficient infection of these cells by the Puumula virus (PUUV) and HTNV occurs from the apical side only, while ANDV and the Black Creek Canal virus (BCCV) can infect epithelial cells from both surfaces [106,107,108]. Additional cellular factors may bring hantaviruses to the appropriate side of the cell for entry. The decay-accelerated factor (DAF or CD55), an inhibitor of the complement cascade, seems an interesting candidate. DAF is involved in the apical entry of PUUV and HTNV into polarized cells, and SNV binds to DAF with high affinity [66,109]. More recently, DAF has been shown to participate in the transport of unrelated viruses in polarized cells, from the apical side to the lateral tight junctions, an environment believed to promote virus-receptor interactions that are otherwise inaccessible for viral particles docking on apical surfaces [46,110,111]. Although DAF does not seem to be required for hantavirus infection of non-polarized cells *in vitro* [67,70], the factor might be critical *in vivo*.

It is apparent that hantaviruses, as other unrelated bunyaviruses, can exploit additional entry factors or even use alternative cellular receptors for infection. A 70-kDa cellular protein on β3 integrin-expressing CHO cells has been found to interact with HTNV [71]. However, in the absence of further work, the identity of this factor remains unknown. Ectopic expression of heterodimers between the human β2 and different α chains of integrins was shown to make CHO cells 4–8-times more susceptible to lentiviral particles pseudotyped with the glycoproteins of HTNV [72]. To ascertain the physiological relevance of this finding, it remains to be assessed whether the virus uses β2 integrin to enter cells that naturally express this protein. HTNV was also found to bind to the receptor of the globular head domain of the complement protein C1q (gC1qR) in Vero cells [70]. The role of gC1qR in HTNV infection was confirmed in approaches based either on silencing in Vero cells or on overexpression of the protein in CHO cells [70]. Nevertheless, it is not clear whether gC1qR acts as a virus receptor or merely as a cellular factor that is important for virus entry beyond attachment to cells. Finally, two independent haploid genetic screens and one small interfering RNA (siRNA)-based screen highlighted the importance of host genes related to cholesterol sensing, regulation and biosynthesis for ANDV infection [112,113]. None of the cellular factors whose role in hantavirus infection is described above were mentioned in these screens, and no new candidate receptor was reported [112,113].

## 6. Bunyavirus Uptake

To enter host cells, it is apparent that bunyaviruses rely on the physical uptake of particles into the endocytic cellular machinery. While the number of reports on receptors and subsequent endocytic pathways used by bunyaviruses has increased over the past decade, the transition processes between the extracellular and intracellular stages remain largely uncovered. However, using fluorescently-labeled UUKV and enhanced green fluorescent protein-tagged DC-SIGN, it was possible for the first time to visualize virus-receptor interactions in live cells and to analyze their dynamics [53]. Using this powerful model system, it was possible to observe receptor recruitment to cell-associated virus particles. This confirmed the hypothesis that viruses collect receptors at the site of contact and thus generate a receptor-rich microdomain in the plasma membrane. Such a series of events is arguably a prerequisite for local plasma membrane curvature and receptor-mediated signal transduction, which in turn results in the sorting of viral particles into the endosomal vesicles [46]. Cholesterol and other lipids also play an important role in these mechanisms by promoting the formation of docking sites for specific proteins. Transduction of cells with lentiviral or rhabdoviral particles pseudotyped with ANDV glycoproteins appears to be sensitive to cholesterol depletion [113,114]. Infection by CCHFV and two orthobunyaviruses, Oropouche (OROV) and Akabane, is abolished in cells depleted of cholesterol by methyl-β-cyclodextrin [115,116,117,118].

Sequence motifs in receptors’ cytosolic tails generally define the identity of the endocytic route in which the cargo is taken up. These motifs serve as docking sites for specific adaptor proteins with functions in signaling, endocytic internalization and intracellular trafficking [46]. The cytosolic tail of DC-SIGN carries several motifs, including two leucines (LL), which are critical for the endocytosis of cargo by the lectin [119,120]. When a mutant of DC-SIGN lacking the LL motif in the cytosolic tail was expressed, UUKV still attached to the cells [53,121]. However, viruses were not internalized, and there was no infection, indicating that DC-SIGN serves as an endocytic receptor, not only as an attachment factor. In contrast to DC-SIGN, the endocytic function of L-SIGN was not required for UUKV infection [62]. Similar levels of infection were obtained in cells expressing either the wild-type lectin or its endocytic-defective mutant. This indicates a fundamental distinction in the use of DC-SIGN and L-SIGN by these viruses for entry; *i.e.* DC-SIGN as an endocytic receptor *versus* L-SIGN as an attachment factor. It is not known whether other signal motifs in receptors identified for bunyaviruses have a function in virus internalization and infection.

**Figure 4:**
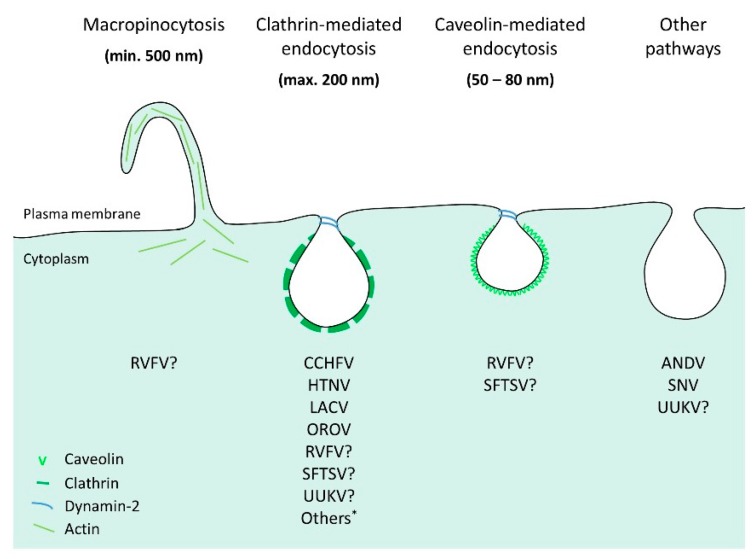
Bunyavirus endocytosis. Endocytic internalization of bunyaviruses into animal cells occurs via various pinocytic pathways, which involve several cellular factors, such as adaptor and coat proteins. A growing body of evidence however indicates that several bunyaviruses use clathrin-mediated endocytosis. * Akabane, Black Creek Canal, California encephalitis, Inkoo, Jamestown, Keystone, Melao, Serra do Navio, Snowshoe Hare, Seoul, Tahyna and Trivittatus viruses. SFTSV: severe fever with thrombocytopenia syndrome virus; UUKV: Uukuniemi virus; RVFV: Rift Valley fever virus; LACV: La Crosse virus; CCHFV: Crimean-Congo hemorrhagic fever virus; ANDV: Andes virus; SNV: Sin Nombre virus; HTNV: Hantaan virus; OROV: Oropouche virus.

Several lines of data suggest that orthobunyaviruses and nairoviruses mainly subvert clathrin-mediated endocytosis (CME) to penetrate and infect cells (Figure 4). Studies based on the use of siRNAs, dominant-negative mutants and chemical inhibitors against adaptor protein 2 (AP2) and clathrin suggest that the orthobunyaviruses Akabane, LACV, OROV and Tahyna, as well as the nairovirus CCHFV depend on CME for infection [115,116,117,118,122,123]. Additional work is still needed to better understand the uptake of phleboviruses. In cells expressing DC-SIGN, electron microscopy pictures did not exclusively show particles of UUKV in clathrin-coated endosomes, and in cells lacking the lectin expression, viral particles could be seen in rare cases associating with clathrin-coated pits and vesicles [53,124]. In addition, clathrin silencing had no significant impact on UUKV infection [124]. Whether RVFV uses clathrin remains unclear. It has been shown that a genetically modified, non-spreading strain of the virus relies on clathrin for successful entry while two independent studies suggest that the RVFV vaccine strain MP12 enters cells both through caveolin-dependent mechanisms and macropinocytosis [125,126,127]. In the case of hantaviruses, the use of CME for virus entry seems to be isolate-specific (Figure 4). A first study based on chemical inhibitors indicates that the HTNV and BCCV, as well as Seoul virus (SEOV) depend on clathrin-mediated uptake, while other studies propose that the SNV and ANDV hijack an alternative endocytic pathway that does not involve clathrin [128,129,130].

Altogether, these reports most likely underline the ability of bunyaviruses to use alternative endocytic routes in a single cell or distinct tissues, as suggested by a growing amount of data obtained for unrelated viruses, such as influenza virus. The divergent endocytic processes by which virus receptors are internalized into the cells, as well as the expression pattern of virus receptors on the cell surface certainly influence the capacity of these viruses to enter one or more endocytic pathways to infect cells and tissues.

## 7. Bunyavirus Intracellular Trafficking

Upon uptake, bunyaviral particles are sorted into vesicles and traffic through the endocytic machinery until reaching the appropriate endosomal compartments for fusion and penetration into the cytosol. Transport from early (EEs) to late (LEs) endosomes is a complex, sensitive cell biological process that is not yet thoroughly understood and involves hundreds of cellular factors with a wide range of functions [131,132]. It is accompanied by major protein and lipid remodeling and concomitant changes in the endosomal luminal milieu [133]. The endosomes provide an environment in which the decreasing pH, from ~6.5 in EEs down to ~5.5–5.0 in LEs and lysosomes, provides a convenient cue for virus activation [134]. Many studies have clearly established the dependence of bunyaviruses on endosomal acidification for infection [54,115,116,117,118,124,125,126,135,136]. Several members from the different genera of the *Bunyaviridae* family are sensitive to extremely low concentrations of lysomotropic weak bases, such as ammonium chloride (in the range of mM), or inhibitors of vacuolar H+ ATPases, such as bafilomycin A1 and concanamycin B (in the range of nM), which all neutralize the endosomal pH.

**Figure 5:**
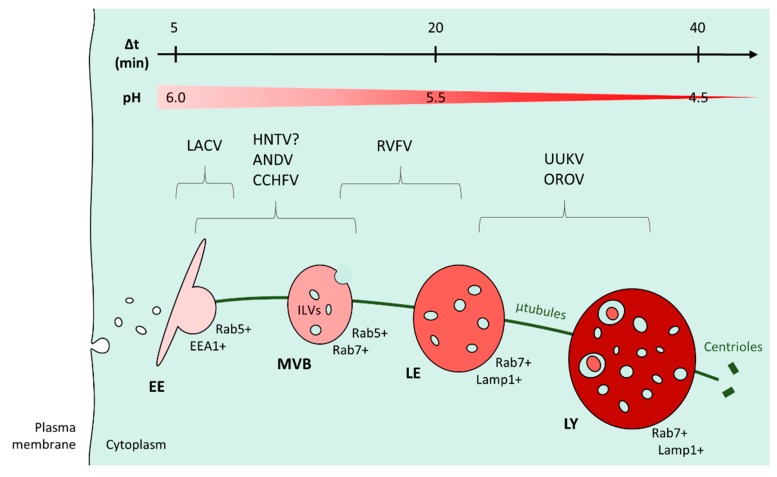
Bunyavirus intracellular trafficking. This figure shows an overview of the different potential locations of bunyavirus penetration. On the top, the scales indicate the time required by a cargo to traffic from the plasma membrane to an organelle (Δt) and the pH inside the endosomes (pH). Abbreviations: EE: early endosome; ILV: intraluminal vesicle; LE: late endosome; LY: lysosome; µtubule: microtubule; MVB: multivesicular body; LAMP: lysosome-associated membrane glycoproteins.

Various data support the view that bunyaviruses transit through EEs during their journey in the endocytic machinery (Figure 5). The expression of dominant negative (DN) and constitutively active mutants against endogenous Rab5, a small GTPase required for the trafficking and maturation of EEs, blocks infection by many bunyaviruses, including UUKV, CCHFV and LACV [117,122,123,124]. Confocal microscopy pictures show the transit of UUKV through Rab5-positive EEs [124], while OROV, CCHFV and HTNV enter vesicles positive for EEA1 [116,117,128], a Rab5 effector protein that exclusively localizes to EEs. An important body of data indicates that bunyaviruses are late-penetrating viruses, a large group of viruses that share dependence on late endosomal maturation for infection [134]. Acid-activated penetration of UUKV and RVFV occurs 20–40 min after internalization, which is compatible with the timing of LE maturation [124,125]. Microtubules are known to drive the trafficking of LEs from the cellular periphery towards the nucleus, a process concomitant with late endosomal maturation. Drug-based inhibitory studies demonstrated that UUKV, CCHFV and some hantaviruses, such as ANDV, BCCV, HTNV and SEOV, require an intact microtubule network for productive infection [124,130,137], which suggests the involvement of LE mobility in virus entry. Viral membrane fusion of UUKV, RVFV, CCHFV and ANDV takes place at pH levels below 6.0, typical for late endosomal vesicles [123,124,125,135,138]. Recently, the phospholipid bis(monoacylglycerol)phosphate (BMP), located in LEs, has been shown to facilitate UUKV membrane fusion [139]. Finally, UUKV and, possibly, HTNV were found in late endosomal compartments [124,128].

While much evidence supports the idea that bunyaviruses penetrate cells from late endosomal compartments and can be confidently considered late penetrating viruses, many of these viruses appear to infect cells independently of active Rab7, the most critical small GTPase for LE trafficking and maturation [140]. Expression of the Rab7 DN mutant has no impact on infection by CCHFV [117,123], and Rab7 does not seem to be essential either for infection by LACV [122]. However, little is known on intracellular trafficking of this latter virus. Though OROV and UUKV were seen in endosomes positive for Rab7, the expression of Rab7 DN mutant T22N has no significant effect on UUKV infection [116,124]. In contrast, the constitutive active mutant of Rab7 seems to promote UUKV infection [124]. Many reasons could explain why Rab7 is dispensable for infection, such as the mislocalization of the Rab mutants and the presence of multiple Rab7 isoforms, among others. Alternatively, viruses might simply escape the endocytic machinery earlier, for example during sorting from EEs to the nascent multivesicular bodies (MVBs), an initial stage in the LE maturation process driven in part by a switch of GTPases from Rab5 to Rab7 [131,133]. This seems to be the case for CCHFV, which has recently been shown to penetrate cells from MVBs [117].

## 8. Bunyavirus-Cell Membrane Fusion

As the ultimate step of the entry program, endocytosed viruses need to cross the membrane of endosomal vesicles to release their genome and accessory proteins into the cytosol. Bunyaviruses accomplish this by membrane fusion, a process intricately coordinated in time and space that is mediated by the viral fusion envelope protein [38,134]. Acidification has been shown to be sufficient to trigger the fusion of mature RVFV, UUKV and ANDV particles in cell-free *in vitro* approaches [113,125,139,141]. While the endosomal pH is a critical signal for triggering the fusion of many enveloped viruses, it is sometimes not sufficient. Among others, interactions with receptors, proteolytic cleavage in viral envelope glycoproteins or specific lipids in the target endosomal membranes may also be required [132]. In addition to the endosomal acidification, SFTSV and LACV penetration seems to involve the cleavage of viral envelope glycoproteins G_N_ and G_C_ by endosomal cathepsin proteases [54]. Using virus-liposome-based assays, the fusion of hantaviruses was shown to also depend on the strict presence of cholesterol in the target membrane [113], while the late endosomal phospholipid BMP was demonstrated to facilitate UUKV fusion [139].

Typically, upon fusion activation, viral fusion proteins undergo multiple conformational changes, target and harpoon the endosome lipid bilayer via the fusion subunit and progressively pull the target and viral membranes through stages of close apposition, hemifusion and fusion pore formation [132,142,143]. The viral material is released through the pore, resulting in the infection of the cell. The Gc glycoproteins show major modifications in their biochemical properties when RVFV, TSWV and ANDV particles are exposed to low-pH buffers [125,141,144]. In the case of ANDV and RVFV, the acid-triggered rearrangement of the G_C_ glycoprotein did not require interactions with target membranes [125,141].

At least three different classes of viral fusion proteins (classes I‒III) have been proposed, each with specific features from structural and mechanistic perspectives [142,143]. The only X-ray structure available for the ectodomain of a bunyavirus glycoprotein is the pre-fusion state of the RVFV G_C_ protein, recently solved at 1.9 Å [34]. The overall fold shows a strong resemblance to class II membrane fusion proteins. Although the degree of amino acid identity is rarely above 30% among the bunyavirus glycoproteins, early bioinformatics predictions and analysis involving peptides and site-directed mutagenesis suggest that the peptide responsible for bunyavirus membrane fusion is carried within the Gc glycoprotein (Figure 2) [35,36,37,135,145,146,147,148].

The ectodomain of class II fusion proteins is composed of three sub-domains (I–III) and is connected to a transmembrane cytosolic tail by a stem region [143,149,150,151]. Exogenous peptides analogous to domain III and the stem region of class II enveloped alpha- and flavi-viruses were successfully employed to block virus fusion and infection [152,153]. It has been demonstrated that such peptides interfere in intramolecular interactions that occur upon acid-activated rearrangement of the viral fusion protein. The presence of these peptides arguably maintains the fusion protein in an intermediate conformation that precedes the post-fusion stage, thereby preventing membrane fusion and virus infection. Similar strategies were used for some bunyaviruses with identical results. Stem peptides that are derived from G_C_ block RVFV infection [154]. Fusion and infection by ANDV and PUUV were also inhibited in the presence of exogenous stem peptides and domain III from G_C_ [155], the sequences of which were predicted from a molecular model for ANDV Gc [36]. In these latter experiments, the fusion was detained at a late stage in the process, before membrane hemifusion [155]. Furthermore, following treatment at low pH, the ANDV G_C_ protein resembled the stable homotrimer post-fusion form of class II fusion proteins [141]. Together, these data suggest that in addition to phleboviruses, hantaviruses also seem to share structural and mechanistic properties with class II enveloped viruses.

Specific histidine residues in fusion proteins, including those from class II proteins, serve as sensors for acidification in endosomal lumen and often define the optimal pH value for virus fusion [156,157,158,159]. The variability of optimal pH can be attributed to the local environment of histidines, which influences their pKa in a range as wide as pH 4.5–7.3 [160]. Such essential histidines have been identified in the RVFV G_C_ by mutational analysis [125], and the optimal pH for penetration has been determined for several bunyavirus isolates using diverse fusion models, from virus glycoprotein-mediated cell-cell fusion assays to virus-liposome and virus-cell-based fusion approaches. Acid-activated viral membrane fusion occurs in a range of pH 5.4–5.6 for RVFV and UUKV [124,125,139], 5.5–6.0 for CCHFV [123], 5.8–6.0 for LACV and California encephalitis virus, another orthobunyavirus [161], 5.8–6.1 for TSWV [144] and at pH ~5.8–5.9 for ANDV [135]. HTNV seems to be the only exception, with a slightly higher activation pH, ~6.3 [136]. Yet, its entry has been observed to also depend on microtubules and LEs, and thus, the cellular factors and location of endosomal escape need more thorough analysis. Ultimately, the major cue for the fusion activation of most bunyaviruses appears to be the endosomal pH.

## 9. Concluding Remarks and Future Perspectives

In this review, we have summarized current knowledge of the early interactions between bunyaviruses and host cells, from virus attachment on the cell surface and uptake to intracellular trafficking and fusion. It is apparent that hundreds of cellular factors with a wide range of functions are involved in bunyavirus entry and penetration into cells. Though each isolate in the family most likely presents specificities, requirements and distinct mechanisms for the very first steps of infection, it seems that many bunyaviruses rely on late, even if partial, endosomal maturation for infection. It is, however, clear that many aspects of the cell biology of bunyavirus endocytosis and penetration require further investigation. High throughput screens involving the use of siRNAs or haploid cells, such as those recently reported for RVFV, UUKV and ANDV [52,112,113,162,163], or ultimately, gene knock-out by the clustered regularly interspaced short palindromic repeats/CRISPR associated protein 9 (CRISPR/Cas9) system may help to identify new cellular factors and processes that are important for bunyavirus endocytosis.

While it is clear that bunyaviruses use many receptors to target and infect a large number of different species and tissues, only a few receptors have been documented in humans and other vertebrates and not a single one in arthropod vectors. Progress will require a detailed cell biological analysis of receptors and the infection process in different types of tissues, not only originating from mammals, but also from other hosts and vectors and all of these under more relevant physiological conditions. Therefore, the combination of new *in vitro* models with *ex vivo* and *in vivo* approaches will certainly allow for novel findings on bunyavirus transmission, entry and spread [164,165,166,167]. Much further work is also needed in the characterization of viral particles originating from different hosts and vectors [167]. The lipid composition of the viral envelope, adaptive mutations in the virus genome and the nature of oligosaccharides in virus glycoproteins arguably influence the identity of first-target cells and the initial stages of infection, including interactions with receptors, sorting into the endocytic machinery and acid-activated fusion.

Ideally, preventing bunyavirus dissemination requires approaches that target the early steps of infection. While identification of the host range is paramount, at the molecular level single inhibitors cannot accurately define a cellular pathway. Perturbants have many side effects or simply impair different processes in cells. Only a combination of well-defined inhibitor profiles, in quantitative and qualitative assays that allow for the monitoring and analysis of the very first minutes of infection, will make the identification of specific cellular factors and mechanisms involved in bunyavirus entry possible.

Virus fusion proteins and uncoating also remain insufficiently characterized given the central role played by acid-activated membrane fusion in bunyavirus penetration. Structural information on the post-fusion form of the G_C_ protein is still missing. It remains to be examined whether other bunyaviruses also rely on class II fusion proteins for penetration or rather additional conformations may be found, as in the *Flaviviridae* family [143,168]. There is evidence that the N-terminal half of the orthobunyavirus G_C_ protein is dispensable for fusion and seems to correspond to an additional functional subunit [145]. A number of outstanding questions also remain regarding the global, highly ordered arrangement and interactions of the G_N_ and G_C_ glycoproteins on the virions [169]. To this extent, the X-ray structures of G_N_ ectodomains are still to be discovered.

These are the keys to broadening our knowledge of bunyaviral dissemination and tissue tropism and, ultimately, helping to develop new anti-bunyavirus strategies. In this regard, all of the factors and mechanisms that have been shown to be involved in virus entry, from virus or cell perspectives, can potentially be used as targets to block the initial steps of transmission and the subsequent spread throughout hosts.

## Figures and Tables

**Table 1:** Potential receptors documented for bunyaviruses.

Transmission	Receptor/Cofactor	Bunyavirus	Genus	Ref.
Arthropod bite	DC-SIGN	SFTSV, UUKV, RVFV	*Phlebovirus*	[50,51,52,53,54,55,61,62,63,64,65]
		LACV	*Orthobunyavirus*	
		CCHFV	*Nairovirus*	
	L-SIGN	RVFV, SFTSV, TOSV, UUKV	*Phlebovirus*	
	NMMHC-IIA	SFTSV		
	Heparan Sulfate	RVFV, TOSV		
	Nucleolin	CCHFV	*Nairovirus*	
	50-kDa protein	TSWV	*Tospovirus*	
Aerosol inhalation	β_3_ integrin	ANDV, SNV, HTNV, PUUV, SEOV, NY-1V	*Hantavirus*	[66,67,68,69,70,71,72]
	β_1_ integrin	PHV, TULV		
	β_2_ integrin	HTNV		
	gC1qR	HTNV		
	DAF	HTNV, PUUV		
	70-kDa protein	HTNV		

DC-SIGN: Dendritic Cell-Specific Intercellular adhesion molecule-3-Grabbing Non-integrin; L-SIGN: Liver-Specific Intercellular adhesion molecule-3-Grabbing Non-integrin; NMMHC-IIA: non-muscle myosin heavy chain IIA; DAF: decay-accelerated factor; SFTSV: severe fever with thrombocytopenia syndrome virus; UUKV: Uukuniemi virus; RVFV: Rift Valley fever virus; LACV: La Crosse virus; CCHFV: Crimean-Congo hemorrhagic fever virus; TOSV: Toscana virus; TSWV: tomato spotted wilt virus; ANDV: Andes virus; SNV: Sin Nombre virus; PUUV: Puumala virus; SEOV: Seoul virus; NY-1V: New York-1 virus; PHV: Prospect Hill virus; TULV: Tula virus; HTNV: Hantaan virus.

## Abbreviations

The following abbreviations are used in this manuscript:

ANDV	Andes virus
BCCV	Black Creek Canal virus
BMP	phospholipid bis(monoacylglycerol)
CCHFV	Crimean-Congo hemorrhagic fever virus
CME	clathrin-mediated endocytosis
DAF	decay-accelerated factor
DC	dendritic cell
DC-SIGN	dendritic cell-specific intercellular adhesion molecule 3-grabbing non-integrin
DN	dominant negative
EE	early endosome
ER	endoplasmic reticulum
GAG	glycosaminoglycan
HTNV	Hantaan virus
HUVEC	human umbilical vein endothelial cell
LACV	La Crosse virus
LE	late endosome
LL	di-leucine
L-SIGN	liver/lymph node-specific intercellular adhesion molecule 3-grabbing non-integrin
MVB	multivesicular body
NMMHC-IIA	non-muscle myosin heavy chain IIA
NY-1V	New York-1 virus
OROV	Oropouche virus
PHV	Prospect Hill virus
PSI	plexin-semaphorin-integrin
PUUV	Puumula virus
RNP	ribonucleoprotein
RVFV	Rift Valley fever virus
SEOV	Seoul virus
SFTSV	severe fever with thrombocytopenia syndrome virus
siRNA	small interfering RNA
SNV	Sin Nombre virus
TOSV	Toscana virus
TSWV	tomato spotted wilt virus
UUKV	Uukuniemi virus
